# Health and Sociodemographic Differences between Individual and Team Sport Participants

**DOI:** 10.3390/sports7060150

**Published:** 2019-06-21

**Authors:** Jennifer R. Pharr, Nancy L. Lough, Mary Angela Terencio

**Affiliations:** 1 School of Public Health, University of Nevada, Las Vegas, NV 89154 USA; 2 College of Education, University of Nevada, Las Vegas, NV 89154, USA; nancy.lough@unlv.edu; 3 School of Allied Health, University of Nevada, Las Vegas, NV 89154, USA; terencio@unlv.nevada.edu

**Keywords:** individual sport, team sport, chronic diseases, Behavioral Risk Factor Surveillance System

## Abstract

Physical activity (PA) has been widely recognized as an avenue to improve health. Researchers have also found better health outcomes among adults who participate in sport when compared to adults who participate in other forms of PA. However, little is known about the health differences between those who participate in individual versus team sport. The purpose of the study was to identify differences in chronic diseases, conditions, or risk factors between individual and team sport participants. This study was a secondary analysis of data from the national Behavioral Risk Factor Surveillance System survey conducted in 2017. PA that was identified as sport was further categorized as an individual or a team sport. Odds and adjusted odds ratios for chronic diseases based on sport category were calculated using logistic regression. There were significant differences in all sociodemographic characteristics between the groups. Those who participated in team sport did so for more minutes and at a higher intensity and were less likely to report several chronic diseases/conditions. However, after controlling for sociodemographic differences between groups, only depression, general health, and smoking remained significant. The social aspect of team sport may be protective against depression but may also influence unhealthy behaviors such as smoking.

## 1. Introduction

Being physically active provides a host of health benefits, in both adult life and childhood. Compared to people who are inactive, those who engage in physical activity (PA) have reduced risk for several chronic diseases and conditions including diabetes, high cholesterol, high blood pressure, obesity, cardiovascular disease, some cancers, and depression [1,2,3,4,5,6]. Additionally, people who are physically active have improved bone density, lung function, mood, and sense of well-being when compared to their inactive peers [2,3,4,7]. Research has also established a dose response between PA and health, with those who exercise at a higher intensity achieving better health outcomes than people who exercise at a lower intensity [8,9,10]. The intensity of PA is measured as metabolic equivalents (METs). METs are the energy cost of an activity and the calories per kilogram of body weight per hour (kcal/kg/h) of an activity. The energy cost of rest is equal to one MET. MET values have been established for different forms of PA, and these values are used to compare the intensity of different activities to rest. As an example, the MET value of playing competitive soccer is 10, meaning that playing competitive soccer requires 10 times more energy than resting.

Building on this knowledge, recent research has examined the health benefits of different categories of PA including: sport, conditioning exercise, recreation, and household tasks [11,12]. This research has found that, for both men and women, those who participate in sport achieve a higher MET level and have reduced risk for many chronic diseases and conditions including high cholesterol, high blood pressure, diabetes, obesity, and cardiovascular disease. Additionally, those who participate in sport are less likely to smoke, report depression, or poor general health when compared to the other three PA categories [11,12].

Sport can be further categorized as individual sport or team sport. Team sport includes interactions between players on the same side or team that are central to the game’s outcome (e.g., soccer, basketball, volleyball). Individual sport includes no such interaction (e.g., swimming, running, cycling) [13]. Research has been conducted to determine differences between those who participate in individual compared to team sport with regard to personality differences and traits; anxiety, depression, and self-confidence; sport addiction; alcohol consumption; and injury risks [14,15,16,17,18,19,20,21,22]. However, no research was identified that explored potential differences in chronic diseases or conditions between these two groups.

Previous research has found that men and women in the United States of America (USA) who participate in sport as compared to other forms of PA have reduced risks for chronic diseases and conditions [11,12]. Additionally, previous research has found differences between those who participate in individual and team sport for things other than chronic diseases or conditions (e.g., self-confidence or positive personality traits). To further our understanding of the intersection of sport, physical activity, and health by adding to this field of research, the purpose of this study was to examine the relationship between individual and team sport participation and health among adults in the USA. Our goal was to see if there were differences in chronic diseases or conditions and risk factors between people who participate in either individual or team sport. Additionally, we aimed to determine if there were differences in MET level, time spent doing sport, or meeting the recommended amounts of PA between the two groups. Because there are differences between those who participate in individual and team sport for things other than chronic diseases or conditions, our hypotheses were:

1. There will be differences in chronic diseases or conditions and risk factors between those who report participating in individual sport compared to team sport and the differences will remain after controlling for sociodemographic characteristics including: sex, age, income, marital status, education, and race/ethnicity.
2. There will be differences in meeting the recommended amount of exercise per week and MET levels between those who participate in individual versus team sport.

## 2. Materials and Methods

For this analysis, we used the same methodology that we used for our studies of the health benefits of sport participation for women and men in the USA which utilized the 2013 and 2015 Behavioral Risk Factor Surveillance System (BRFSS) data, respectively [11,12].

### 2.1. Study Design and Setting

This study was a secondary analysis of data from the 2017 cross-sectional Behavioral Risk Factor Surveillance System (BRFSS) study. Of all health-related surveys of adults conducted in the USA, the BRFSS is the largest. The BRFSS has been conducted annually since 1984 and is a collaboration between the Centers for Disease Control and Prevention (CDC) and the 50 states and three territories in the USA [23]. 

### 2.2. Participants

The BRFSS is a random-digit dial telephone survey which includes both cellular and landlines. Participants include non-institutionalized adults over the age of 18 years [23]. In 2017, 450,016 people participated in the survey. Disproportionate stratified sampling is used to provide an adequate sample size for smaller demographic areas [23]. Data are also weighted for population attributes and non-response [23]. For additional information about the BRFSS weighting, sampling, and survey administration, please visit https://www.cdc.gov/brfss.

### 2.3. BRFSS Survey and Variables

All participants in the BRFSS survey are asked the questions from the core component of the survey, which includes questions about their sociodemographic characteristics, chronic diseases, health promoting behaviors, and health risk behaviors. Participants are asked an extensive set of questions about their exercise behaviors in odd years [24]. The initial question in the exercise module is, “During the past month, other than your regular job, did you participate in any physical activities such as running, calisthenics, golf, gardening, or walking for exercise?” [24]. If a participant answers “yes” to this question they are then asked follow-up questions from the exercise module. The next question in the exercise module is, “What type of physical activity or exercise did you spend the most time doing the past month?” [24]. Participants can select only one activity or exercise for their response to this question, and there are 76 different activities/exercises. The next two questions in the exercise module are, “How many times per week or per month did you take part in this activity during the past month?” followed by, “And when you took part in this activity, for how many minutes or hours did you usually keep at it?” [24].

Based on responses to questions in the exercise module, the CDC applies an algorithm to determine whether or not participants met the recommended amount of aerobic exercise. The results are categorized as “Meeting aerobic recommendations” (150+ min of moderate aerobic activity) or “Not meeting aerobic recommendations” (0–149 min of aerobic exercise) [24]. The CDC also assigns a MET value based on the answers to the questions in the exercise module.

Using the answers to the questions in the exercise module (activity, duration, and frequency), the CDC assigns participants to the PA categories of highly active, active, insufficiently active, or inactive. The PA categories include highly active (300 min of moderate aerobic activity or 150 min of vigorous aerobic exercise), active (150–300 min of moderate aerobic activity or the vigorous equivalent), insufficiently active (11–149 min of moderate aerobic activity), and inactive (no PA) [24]. The BRFSS exercise questions and categories have been found to have high validity (compared with other surveys, participant logs, accelerometers, or other PA measures) and reliability (test/retest comparisons) [25].

Participants provide sociodemographic data which includes marital status, sex, age, education, employment, income, and race/ethnicity. They are asked about chronic diseases or conditions including: cardiovascular disease (CVD), heart attack, stroke, high cholesterol, high blood pressure, diabetes, overweight/obesity, asthma, skin cancer, other cancers, depression, arthritis, chronic obstructive pulmonary disease (COPD), and kidney disease. Answers to the chronic disease or conditions questions are dichotomized as ‘yes’ or ‘no’, with ‘refused to answer’ or ‘I don’t know’ considered missing. Additionally, they are asked about risk factors including smoking and binge drinking; these are dichotomized as well.

Two researchers had previously reviewed the 76 different activities/exercises and independently placed them into four predetermined PA categories of: sport, conditioning exercise, household tasks, and recreation. Activities that were identified as sport were further categorized as individual or team sport based on the definition of team and individual sport provided in the introduction. Individual sports include: badminton, cycling, boxing, golf, American handball, racquetball, running, rock climbing, tennis, and wrestling. Team sports include: basketball, rowing (competitive), hockey, lacrosse, rugby, soccer, softball/baseball, squash, touch football, and volleyball. For this study, people who answered “no” to the initial exercise question, who refused to answer the second exercise question, or those whose physical activity was classified as something other than sport were excluded from our analysis.

### 2.4. Data Analyses

Statistical analyses of sociodemographic characteristics and chronic diseases or conditions and risk behaviors by sport type were performed using SAS Version 9.3 (SAS Institute Inc., Cary, NC, USA). Weighted descriptive analyses were performed to describe the sociodemographic characteristics by marital status, sex, age, education, employment, income, and race/ethnicity. Rao X^2^ tests were performed using PROC SURVEYFREQ to determine statistically significant differences in sociodemographic characteristics, PA categories (highly active, active, insufficiently active, or inactive), and meeting aerobic recommendation by sport type. Additionally, the mean number of minutes and the mean METs along with the 95% confidence interval (CI) were calculated using PROC SURVEYMEANS. Odds ratios and adjusted odds ratios for chronic diseases or conditions by sport type were calculated using logistic regression through the PROC SURVEYLOGISTIC function. Because there were significant differences between the two groups for all sociodemographic characteristics, we controlled for these variables in our adjusted odds ratios.

Because this study was a secondary data analysis of de-identified data, it was excluded from review by the University of Nevada, Las Vegas Institutional Review Board.

## 3. Results

### 3.1. Sociodemographic Characteristics

There were significant differences in all sociodemographic characteristics (Table 1). People who participated in individual sport were more likely to be married, college graduates, older, White, in the highest income bracket, and out of the labor force (retired). People who participated in team sport were more likely to be younger, single, men, Black or Hispanic, and employed. 

### 3.2. Odds and Adjusted Odds Ratios for Chronic Diseases or Conditions and Risk Factors

People who participated in team sport had significantly lower odds for reporting several chronic diseases and conditions including: high cholesterol, high blood pressure, skin cancer, other cancer, heart attack, cardiovascular disease, arthritis, depression, and diabetes (Table 2). They were also less likely to report being in fair to poor health as opposed to excellent to good health. However, those who participated in team sport were significantly more likely to report being overweight or obese, binge drinking, or being a current smoker. After controlling for sociodemographic characteristics, the only variables that remained significant were general health (fair to poor), depression, and currently smoking.

### 3.3. Physical Activity Categories by Sport Type

People who participated in team sport were significantly more likely to be categorized as highly active or active when compared to people who participated in individual sport (Table 3). Additionally, people who participated in team sport were significantly more likely to have met the recommendations for aerobic exercise. These rates may be due to people who participated in team sport reporting doing so for a longer period of time and at a higher intensity (Table 4).

## 4. Discussion

Previous research has established the health benefits of physical activity and sport participation [2,4,11,12]. However, there is a paucity of research examining the health benefits of individual or team sport participation. We found that individuals who participated in team sport as their primary source of physical activity had decreased risk for many chronic diseases or conditions including high blood pressure, high cholesterol, diabetes, skin cancer, other cancers, arthritis, depression, heart attack, and cardiovascular disease. However, none of these remained significant after adjusting for demographic characteristics with the exception of depression and fair-to-poor general health. Our univariate findings may be due to individuals who participate in individual sport being in a higher age bracket, which increases the likelihood of being diagnosed with a chronic disease or condition. A report from the CDC shows that 78% of adults in the USA aged 55 years and older have at least one chronic disease [26].

Participation in team sports can have a positive impact that affects an individual’s well-being. We found that those who participated in team sport as their primary source of physical activity had a lower risk for depression. Other researchers have found that those who engage in sports that emphasize collaboration, camaraderie, and team effort report higher levels of intrinsic motivation and a sense of belonging and social connectedness [27,28]. These athletes have opportunities to be active in a social context and can form meaningful relationships and peer support, increasing their mental health scores [29]. Playing as a team decreases symptoms of depression and, compared to individual sports, has greater effects in reducing it, especially if they have been playing for a longer period of time [30,31,32]. Participating in team sport is also associated with the lowest mental health burden in comparison to individual sport [22]. These benefits can be seen across the lifespan from adolescence to older populations [22,28,31,32]. However, depending on the competitive nature and intensity of the sport, there may be higher levels of anxiety and depression, especially if the level of skill of the participant does not match the challenge of the sport [21]. Moreover, factors such as overtraining, injuries, and failure in competition can negatively affect athletes and their mental health, but this is more prevalent in those who participate in individual sports [19].

Previous research has found that people who participate in sport as a form of PA are less likely to smoke when compared to people who engage in other forms of PA or who are physically inactive [11,12]. However, few studies have examined the relationship between sport participation type and smoking. Those that have examined this relationship have identified a greater risk of smoking among those who participated in team sport compared to individual sport [19,33,34,35]. Additionally, other research has found that those who participate in team sport have greater risk of problematic alcohol consumption and cannabis use when compared to those who participate in individual sport [36,37]. These unhealthy behaviors found among team sport participants may be due to socialization, peer pressure, or pressure to perform found more often in team sport compared to individual sport [36]. We found that people who participated in team sport were more likely to be current smokers when compared to those who participated in individual sport, and this finding remained after controlling for sociodemographic variables. We also found greater odds for binge drinking in the team sport group; however, that finding was not significant once we adjusted for demographic characteristics.

As with any study, this study has limitations. Because the BRFSS is a cross-sectional study, causation cannot be determined [38]. There was a possibility of self-report bias as participants may also have over- or underreported information if they perceived the response to be socially desirable [39]. Additionally, we were only able to categorize and analyze the sport that was participated in the most. Some participants may have engaged in both team and individual sports; however, the second sport category was not included in this analysis.

## 5. Conclusions

An emerging field of research is examining the intersection of sport, physical activity, and health. Previous research has found that sport participation is associated with reduced risk for many chronic diseases or conditions. This study furthered that research by examining chronic diseases or conditions by sport type—individual or team. The sociodemographic characteristics of the two groups were different and, after controlling for sociodemographic differences, only depression, general health, and smoking remained significant. The social aspect of team sport may be protective against depression but may also influence unhealthy behaviors such as smoking.

## Figures and Tables

**Table 1:** Sociodemographic characteristics by sport type—percentages and overall Rao X^2^.

Variable	Variable	Total N 39,281	Individual Sport N (%) 34,907 (88.9)	Team Sport N (%) 4374 (11.1)	Overall X^2^ and *p*-Value
Sex	Male	68.0%	64.8%	87.4%	297.1, *p* < 0.01
	Female	32.0%	35.2%	12.6%	
Marital status	Married	44.3%	45.8%	35.3%	110.1, *p* < 0.01
	Single	40.1%	38.5%	50.1%	
	Divorced	6.7%	7.0%	4.4%	
	Widowed	1.6%	1.8%	0.6%	
	Separated	1.4%	1.3%	2.1%	
	Partnered	5.9%	5.6%	7.4%	
Educational Attainment	Did not graduate HS	8.8%	7.8%	14.7%	143.7, *p* < 0.01
	High school graduate	24.3%	23.0%	32.0%	
	Some college	31.1%	31.2%	30.2%	
	College graduate	35.9%	38.0%	23.1%	
Age (years)	18–24	28.9%	27.4%	38.2%	235.9, *p* < 0.01
	25–34	25.3%	24.8%	28.5%	
	35–44	18.0%	17.8%	19.6%	
	45–54	12.5%	13.2%	8.7%	
	55–64	8.2%	9.0%	3.3%	
	64–74	4.8%	5.4%	1.2%	
	75+	2.2%	2.5%	0.4%	
Race/Ethnicity	White	59.4%	61.5%	46.2%	
	Black	9.9%	9.0%	15.4%	
	Other	9.6%	9.9%	8.3%	
	Multi	1.5%	1.5%	1.3%	
	Hispanic	19.5%	18.1%	28.8%	
Income	<10 K	4.5%	4.2%	6.4%	61.0, *p* < 0.01
	10–25 K	16.3%	15.4%	22.5%	
	25–50 K	20.5%	20.3%	21.9%	
	50–75 K	13.8%	13.8%	14.1%	
	>75 K	44.9%	46.4%	35.1%	
Employment	Employed	69.4%	68.8%	73.7%	16.8, *p* < 0.01
	Unemployed	4.9%	4.8%	5.4%	
	OLF	24.1%	24.9%	19.5%	
	Unable to work	1.5%	1.6%	1.4%	

HS = high school, OLF = out of labor force.

**Table 2:** Odds and adjusted ratios for chronic diseases or conditions and risk factors with individual sport as the reference.

Variable	OR	95% CI	AOR	95% CI
General Health (fair–poor)	**0.70**	**0.62–0.79**	**0.81**	**0.70–0.93**
High Blood Pressure	**0.80**	**0.68–0.94**	0.93	0.76–1.12
High Cholesterol	**0.68**	**0.57–0.81**	0.96	0.78–1.18
Heart Attack	**0.44**	**0.27–0.72**	0.83	0.46–1.50
CVD	**0.55**	**0.39–0.76**	0.85	0.59–1.23
Stroke	0.71	0.41–1.25	0.92	0.52–1.61
Asthma	1.061	0.90–1.25	0.99	0.82–1.21
Skin Cancer	**0.30**	**0.21–0.52**	0.82	0.50–1.34
Other Cancers	**0.39**	**0.25–0.59**		
COPD	0.97	0.68–1.38	1.21	0.79–1.85
Arthritis	**0.59**	**0.48–0.72**	0.98	0.77–1.25
Depression	**0.60**	**0.51–0.72**	**0.69**	**0.57–0.85**
Kidney Disease	0.95	0.47–1.89	1.24	0.52–2.97
Diabetes	**0.58**	**0.42–0.79**	0.90	0.63–1.28
Overweight/Obese	**1.19**	**1.05–1.35**	1.11	0.95–1.28
Current Smoker	**1.56**	**1.32–1.84**	**1.25**	**1.02–1.53**
Binge Drinking	**1.24**	**1.08–1.41**	1.11	0.95–1.30

Bolding = significant odds ratios; CI = confidence interval; OR = odds ratio; AOR = adjusted odds ratio; CVD = cardiovascular disease; COPD = chronic obstructive pulmonary disease.

**Table 3:** Exercise amounts by sport type.

Variable	Variable	Individual Sport %	Team Sport %	X^2^ and *p*-Value
Physical Activity Categories	Highly active	47.4%	50.4%	11.2, *p* = 0.01
	Active	26.7%	28.3%	
	Insufficiently active	24.9%	20.4%	
	Inactive	1.0%	1.0%	
Aerobic Exercise Recommendations	Met aerobic recommendations	74.3%	78.8%	11.3, *p* < 0.01
	Did not meet aerobic recommendations	25.7%	21.2%	

**Table 4:** Exercise minutes and metabolic equivalence (MET) by sport type.

Variable	Individual Sport Mean (95% CI)	Team Sport Mean (95% CI)
Minutes of Exercise	230.1 (224.7–235.5)	293.2 (273.1–313.3)
MET	60.7 (60.4–60.9)	64.1 (63.4–64.7)
